# Adrenomedullin-RAMP2 Enhances Lung Endothelial Cell Homeostasis Under Shear Stress

**DOI:** 10.3390/cells15020152

**Published:** 2026-01-14

**Authors:** Yongdae Yoon, Sean R. Duffy, Shannon E. Kirk, Kamoltip Promnares, Pratap Karki, Anna A. Birukova, Konstantin G. Birukov, Yifan Yuan

**Affiliations:** 1 Department of Anesthesiology, University of Maryland School of Medicine, Baltimore, MD 21201, USA; yongdae0611@gmail.com (Y.Y.); sean.duffy@som.umaryland.edu (S.R.D.); skirk@som.umaryland.edu (S.E.K.); kpromnares@som.umaryland.edu (K.P.); kbirukov@som.umaryland.edu (K.G.B.); 2 Pulmonary and Critical Care Medicine, University of Maryland School of Medicine, Baltimore, MD 21201, USA; pkarki@som.umaryland.edu (P.K.); abirukova@som.umaryland.edu (A.A.B.); 3 Section of Pulmonary, Critical Care and Sleep Medicine, Department of Medicine, Yale School of Medicine, New Haven, CT 06519, USA

**Keywords:** lung vasculature, endothelium, paracrine signaling, bioengineering

## Abstract

Analysis of pulmonary vascular dysfunction in various lung pathologies remains challenging due to the lack of functional ex vivo models. Paracrine signaling in the lung plays a critical role in regulating endothelial maturation and vascular homeostasis. Previously, we employed single-cell RNA-sequencing (scRNAseq) to systematically map ligand–receptor (L/R) interactions within the lung vascular niche. However, the functional impact of these ligands on endothelial biology remained unknown. Here, we systematically evaluated selected ligands in vitro to assess their effects on endothelial barrier integrity, anti-inflammatory responses, and phenotypic maturation. Among the top soluble ligands, we found that adrenomedulin (ADM) exhibited superior barrier enhancing effect on human pulmonary endothelial cell monolayers, as evidenced by electrical cell impedance sensing (ECIS) and XperT assays. ADM also exhibited anti-inflammatory properties, decreasing ICAM1 and increasing IkBa expression in a dose-dependent manner. Perfusion is commonly used in bioengineered vascular model systems. Shear stress (15 dynes/cm^2^) alone increased endothelial characteristics, including homeostatic markers such as *CDH5*, *NOS3*, *TEK*, and *S1PR1*. ADM treatment maintained the enhanced level of these markers under shear stress and further improved anti-coagulation by increasing *THBD* and decreasing *F3* expression and synergistically enhanced the expression of the native lung aerocyte capillary endothelial marker *EDNRB*. This effect was completely attenuated by a blockade of ADM receptor, RAMP2. Together, these findings identify ADM/RAMP2 signaling as a key paracrine pathway that enhances vascular barrier integrity, anti-inflammatory phenotype, and endothelial homeostasis, providing a framework for improving the physiological relevance of engineered vascular models.

## 1. Introduction

Pulmonary vascular disease is a growing global health concern and a significant cause of morbidity and mortality [1]. This condition is characterized by microvascular remodeling and rarefaction and is primarily attributed to pathological changes in the vascular endothelium [2]. Current in vitro systems, including organ-on-chips and organoid platforms, have advanced our ability to model vascular systems by incorporating differentiated or mature endothelial cells into hydrogel or polydimethylsiloxane (PDMS)-based perfused systems. These models replicate aspects of physiological flow and intercellular communication [3,4,5,6], providing valuable insights into disease mechanisms. However, these platforms fall short of replicating the complex physiology of the human lung vasculature and lack critical microenvironmental cues in the vascular niche that is essential to alveolar endothelial function, including paracrine signaling, matrix substrate components, and correct substrate stiffness. As a result, no in vitro system can recapitulate native pulmonary vascular cellular phenotypes and physiological functions over extended periods in a controlled manner.

During normal physiological conditions, local cell–cell interactions among endothelial, epithelial, stromal, and immune cell populations within the lung vascular niche can jointly facilitate endothelial homeostasis and protect vasculature from injury. For example, cell–cell crosstalk paracrine factors, including angiopoietin-1 (Ang-1), and slit guidance ligand 2 (Slit2), are secreted in the vascular niche to enhance barrier function and reduce inflammation, preventing the influx of cells and plasma from the bloodstream into the local tissues (reviewed in [7]). To investigate these crosstalk signals under homeostatic conditions, we previously employed single-cell RNA-sequencing (scRNA-seq) to identify cellular populations in human lungs and to characterize signaling molecules secreted by alveolar epithelial, mesenchymal, and immune cells that may be sensed by endothelial cells [8]. We mapped putative ligand–receptor (L/R) interactions within and between cell types and assessed the significance of these interactions using the NicheNet database [8]. From this analysis, we identified a subset of L/R pairs, including *ADM/RAMP2*, *SLIT2/ROBO4*, *BMP5/BMPR2*, that are highly enriched in the lung vascular niche and potentially interact with receptors expressed by vascular endothelial cells and contribute to vascular homeostasis. In this study, we assessed the impact of these top ligands on endothelial cells and tested their ability in regulating vascular cellular phenotypes and physiological functions in the in vitro vascular model systems.

The endothelium lining the blood vessels is highly sensitive to hemodynamic forces that act at the vascular luminal surface in the direction of blood flow (reviewed in [9]). Current bioengineered vascular models, including organ-on-chips, and organoid-on-chips, have incorporated perfusion into their system to simulate native biophysical conditions [10,11]. It was proposed that endothelial cells have their preferred fluid shear stress, or ‘set point’ [12]. Upon exposure to 16 h of laminar shear stress ranging from 2 to 60 dynes/cm^2^, human umbilical vein endothelial cells (HUVECs) aligned in the direction of the flow and anti-inflammatory and endothelial quiescence pathways were activated in the range of 10 to 20 dynes/cm^2^, but cells were misaligned or oriented perpendicularly (against the flow direction), and inflammation and coagulation signaling was activated outside this range ([12] and reviewed in [13]). In this study we investigated the impact of the top ligands involved in paracrine signaling in the intact lung, on cell–cell junction, anti-inflammatory response, anti-coagulation, and native cellular phenotypes in both static and dynamic flow conditions at a shear stress of 15 dynes/cm^2^ in order to identify key players preserving pulmonary endothelial homeostasis that may improve the fidelity of the engineered in vitro models of the pulmonary vasculature.

## 2. Methods

### 2.1. Materials

Adrenomedullin (ADM, #24889), Secretin (24990), and Transforming growth factor beta1 (TGF-β1) from Cayman Chemical (Ann Arbor, MI, USA); Slit homolog 2 (SLIT2, # 8616-SL), Semaphorin 6D (Sema6D, 2095-S6), Pleiotrophin (252-PL), vascular endothelial growth factor A (VEGFA, BT-VEGF), C-C motif chemokine ligand-19 (CCL19, 361-MI/CF), CXC Motif Chemokine Ligand-6 (CXCL6, 333-GC/CF), and TNF-α (# 10291-TA) from R&D Systems (Minnneapolis, MN, USA); Angiopoietin-1 (ANGPT1, #923-AN) and Bone morphogenic protein 5 (BMP5, # 615-BMC-020) and BMP3 (NBP2-61322) from Novus Biologicals (Centennial, CO, USA); and BMP7 (595602), BMP6 (595502), BMP4 (59202), and BMP-2 (767302) were purchased.

### 2.2. Cell Culture

Human pulmonary artery endothelial cells (HPAECs) were purchased from Lonza (Basel, Switzerland), and cells from two different donors were used for all experiments. The cells between passage 6–7 were placed on fibronectin 1 µg/cm^2^ coated plates and were incubated in EGM-2 endothelial cell growth medium-2 bulletkit from Lonza under a cell culture incubator at 37 °C with 5% CO_2_ and a humidified atmosphere.

### 2.3. Electrical Cell–Substrate Impedance Sensing

Electrical cell–substrate impedance sensing (ECIS) assays were performed according to the manufacturer’s instructions. Briefly, ECIS Cultureware plates (e.g., 96W20idf for static conditions or 1F8 × 10E chips for shear stress) from Ibidi (Grafelfing, Germany) were incubated with L-cysteine (10 mM) and then coated with fibronectin (1 µg/cm^2^). For static conditions, cells were incubated for 2 days until a stable baseline impedance was achieved. Ligands were then added to the cells, and changes in impedance were measured to determine endothelial cell permeability. For shear stress conditions, cells were incubated for 1 day after attachment. A unidirectional shear stress of 15 dynes/cm^2^ was applied to the cells, and ADM was added for one more day with continuous impedance measurement.

### 2.4. Expression Permeability Test Assay

To verify endothelial cell permeability to macromolecules, expression permeability test (XperT) assay was performed, as previously described [14]. In brief, cells were seeded on plates coated with biotinylated collagen type I (0.25 mg/mL) dissolved in a 0.1 M bicarbonate, pH 8.3 solution. After ligand treatment, the cells were incubated with FITC-avidin (25 µg/mL) for 2 min. Unbound FITC-avidin was removed by washing with PBS. The fluorescence signal was then either visualized using an EVOS microscope (Thermo Fisher Scientific, Waltham, MA, USA) or measured with a SpectraMax^®^ iD5 microplate reader (Molecular Devices, San Jose, CA, USA).

### 2.5. Western Blot

Cells were lysed with 2× Laemmli sample buffer (#1610737, Bio-Rad, Hercules, CA, USA). The lysates were boiled for 5 min and then subjected to SDS-polyacrylamide gel electrophoresis (SDS-PAGE). Proteins were transferred onto a polyvinylidene difluoride (PVDF) membrane (Bio-Rad). The membrane was blocked with 5% skim milk or 5% BSA in Tris-HCl-buffered saline containing 0.05% Tween 20 (TBST) for 30 min. It was then incubated with primary antibodies against ICAM-1 (sc-8439) and GAPDH (sc-47724) from Santa Cruz Biotechnology (Santa Cruz, CA, USA), as well as IκBα (#4814), NF-κB p65 (#3032), and phospho-NF-κB p65 Ser536 (#3031) from Cell Signaling Technology (Danvers, MA, USA). Primary antibody incubation was performed overnight at 4 °C. Following primary antibody incubation, the membrane was incubated with horseradish peroxidase (HRP)-conjugated secondary antibodies (7074S and 7076S, Cell Signaling Technology) for 2 h. After washing the membrane three times with TBST, the protein bands were visualized using a ChemiDoc XRS+ System (Bio-Rad) after treatment with SuperSignal West Pico PLUS or Atto Ultimate sensitivity chemiluminescent substrate (34578 or A38556, Thermo Fisher Scientific). The band image was processed with Image Lab 6.1 software (Bio-Rad).

### 2.6. qPCR

Total RNA was purified using the RNeasy Plus Mini Kit (#74136, Qiagen, Hilden, Germany) according to the manufacturer’s instructions. Complementary DNA (cDNA) was synthesized from the purified RNA using an iScript cDNA Synthesis Kit (#1708890, Bio-Rad). For real-time PCR, the cDNA was mixed with gene-specific primers and PerfeCta SYBR Green FastMix (Quantabio, Beverly, MA, USA). The reactions were run on a CFX Opus 384 Real-Time PCR System (Bio-Rad). The primer sequences are listed in Table 1. Relative mRNA expression was quantified using the 2^−(△△Ct)^ method.

### 2.7. Enzyme-Linked Immunosorbent Assay (ELISA)

The levels of soluble intercellular adhesion molecule-1 (ICAM-1) in the culture medium were measured using a human ICAM-1 ELISA kit (#DY720) and a Clear Microplate (#DY990) from R&D Systems (Minneapolis, MN, USA). Cells were treated with TNF-α (1 ng/mL) for 30 min, followed by treatment with ADM (100 ng/mL) for 6 h. The assay was performed according to the manufacturer’s instructions. The values were quantified by measuring absorbance at 450 nm using a SpectraMax^®^ iD5 (Molecular Devices, CA, USA).

### 2.8. Shear Stress

To apply a unidirectional shear stress to endothelial cells, we utilized an ibidi pump system (#10902, ibidi, Gräfelfing, Germany). The cells were seeded on µ-Slide 0.4 Luer ibiTreat (#80176, Ibidi) coated with fibronectin (1–2 µg/cm^2^). Following the manufacturer’s instructions, the slides were connected to the pump system. The cells were subjected to 15 dynes/cm^2^ shear stress for 1 day. Subsequently, the medium was carefully replaced with fresh medium with or without ADM (100 ng/mL), and the cells were incubated for an additional day under shear stress at 15 dynes/cm^2^.

### 2.9. Immunofluorescence

For static condition, HPAECs were cultured on glass coverslips coated with fibronectin at 2 µg/cm^2^. For shear stress condition, cells were seeded in a µ-Slide ^0.4^ Luer ibiTreat chip (#80176, Ibidi) with fibronectin coating. Cells in both conditions were fixed with 2% paraformaldehyde in PBS including Ca^2+^ and Mg^2+^ and permeabilized with 0.1% Triton X-100 in PBS for 15 min at RT. Following a 30 min incubation with 5% BSA in PBS, cells were incubated with VE-cadherin antibody (#93467, Cell Signaling Technology) in 5% BSA in PBS at 4 °C overnight. The cells were then labeled by an Alexa Fluor^TM^ Plus 488-conjugated secondary antibody (Invitrogen, Carlsbad, CA, USA) for 1 h at RT. For filamentous actin staining, the cells were incubated with Texas Red-X phalloidin (#T7471, Thermo Fisher Scientific) for 30 min at RT. After DAPI staining (#62248, Thermo Fisher Scientific), the stained cells were mounted by a ProLong glass antifade Mountant. Images were captured using a fluorescent microscope (Eclipse TS2R, Nikon, Tokyo, Japan).

### 2.10. siRNA Transfection

Transfection of RAMP2 (sc-36378) or control siRNA (sc-37007) from Santa Cruz Biotechnology (Santa Cruz, CA, USA) was performed using Lipofectamine RNAiMAX Transfection Reagent (13778-075, Thermo Fisher Scientific, MA, USA) according to the manufacturer’s instructions. Briefly, HPAECs were seeded in fibronectin-coated 6-well plates. Cells were then transfected with either RAMP2 or control siRNA. Following a 24 h incubation, the transfected cells were replated onto ECIS plates for static conditions, ECIS chips for shear stress experiments, or µ-Slide Luer ibiTreat chips for immunofluorescence.

### 2.11. Fluorescence Intensity

Fluorescence intensity of VE-cadherin at cell–cell junction was quantified using Image/Fiji (NIH). For junctional analysis, the VE-cadherin channel was used, and regions of interest (ROIs) were randomly defined along the cell borders of individual cells using a freehand tool. Only clearly identifiable junctional regions were selected. To correct background fluorescence, the mean fluorescence intensity of cytoplasmic regions from the same cells was subtracted from the mean whole-cell fluorescence intensity, and resulting values were used as junction-enriched VE-cadherin intensity.

### 2.12. Statistical Analysis

Data are presented as the mean ± standard deviation (SD) from three independent experiments. Statistical analysis was performed using GraphPad Prism 10 software. Normality of the data was assessed using the Shapiro–Wilk test. For normally distributed data, comparisons between two groups only, an unpaired two-tailed Student’s *t*-test was used, if not, Mann–Whitney U test was used. For analysis involving more than two groups, ordinary one-way ANOVA was performed, followed by Tukey’s multiple comparisons test. For analysis on two independent variables, ordinary two-way ANOVA was used to assess the main effects of each factor and their interaction, with post hoc multiple comparisons conducted using Fisher’s least significant difference (LSD) test. A *p*-value of less than 0.05 was considered statistically significant. * *p* < 0.05; ** *p* < 0.01; and *** *p* < 0.001

## 3. Results

### 3.1. ADM Improves Endothelial Barrier Function

**Figure 1:**
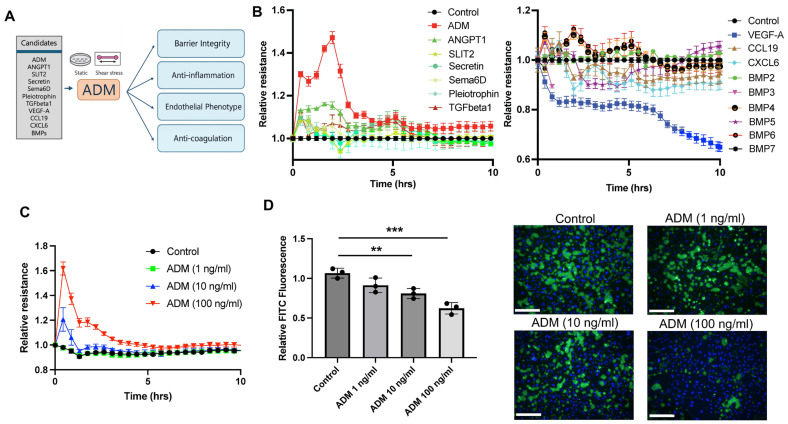
ADM exhibits superior effect on enhancing vascular endothelial barrier function. (**A**) The image presents an overview of experimental flow, including a list of candidate ligands that were selected from a preliminary analysis of ligand/receptor pair between endothelial cells and non-endothelial cells in macrovascular environments. (**B**) Barrier function of HPAEC monolayers was tested by ECIS assay after the cells were treated with the selected ligands (Adrenomedullin (ADM, 100 ng/mL, angiopoietin 1 (ANGPT1, 2 μg/mL), SLIT2 (1 μg/mL), Secretin (500 ng/mL), Sema6D (1 μg/mL), Pleiotrophin (1 μg/mL), TGF-β (100 ng/mL), VEGF-A (100 ng/mL), CCL19 (1 μg/mL), CXCL6 (1 μg/mL), and BMPs (500 ng/mL)). (**C**) Dose-dependent effect of ADM (1–100 ng/mL) on HPAECs was measured using ECIS assay. (**D**) HPAECs were seeded on biotinylated collagen type I (0.25 mg/mL) coated well plates. The cells were pretreated with the indicated ADM concentrations for 1 h, and FITC-conjugated avidin was added for 2 min. The FITC fluorescence was measured, and the image of FITC signal was captured after DAPI staining. The graph is presented as the means ± standard deviation (SD) from three independent experiments. Statistical analysis was performed using one-way ANOVA. White scale bars: 150 μm, ** and *** indicate *p* < 0.01 and *p* < 0.001, respectively.

To determine the impact of native soluble ligands expressed in human lung vascular niche on endothelial homeostasis, we leveraged our L/R pair database previously generated from scRNAseq data of human lungs [8]. We focused our analysis on the top ligands, including ADM, Ang-1, Slit2, Secretin, Sema6D, Pleiotrophin, TGF-β1, VEGF-A, CCL19, CXCL6, BMP2, BMP5, BMP4, BMP6, and BMP7, expressed by alveolar epithelial, mesenchymal, and immune cells, within the vascular niche that could interact with receptors on endothelial cells identified through our previous Connectome analysis (Figure 1A and [8]). Among the ligands tested, we found that both ADM and Ang-1 enhanced endothelial cell barrier integrity, with ADM exhibiting the strongest effect in HPAECs. In contrast, VEGF-A strongly, and CXCL-6, CCL-19, and BMP5 modestly impaired the endothelial barrier (Figure 1B) either through damaging paracellular junction or increased transcytosis, while all other ligands had no or negligible impact on cell–cell junction. Additionally, the XperT and ECIS assays consistently demonstrate ADM increased barrier function in a dose-dependent manner in HPAECs, especially at 10 and 100 ng/mL (Figure 1C,D). These results demonstrate that the soluble ligands identified from native human lung niche might have a distinct impact on endothelial barrier function, and ADM exerts a superior barrier-protective impact as compared to all other tested ligands in vitro cell culture models.

### 3.2. ADM Reduces Inflammatory Responses

**Figure 2:**
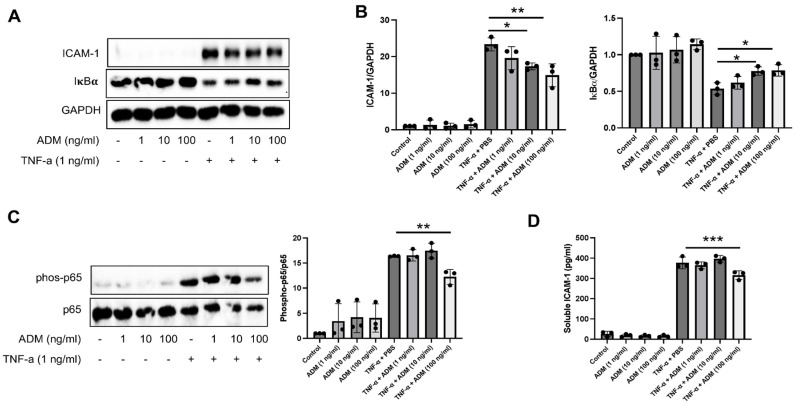
ADM attenuates pro-inflammatory effect of TNF-α. (**A**,**C**) HPAECs were treated with TNF-α (1 ng/mL, 30 min) followed by the ADM addition at indicated concentrations for 5.5 h. The samples were subjected to Western blot (WB) analysis to detect ICAM-1, IκBα, phosphorylated-p65 (S536), total p65, and GAPDH proteins. (**B**) ICAM-1 and IκBα proteins were normalized to GAPDH, and the values were presented as a bar graph. (**C**) Phosphorylated-p65 (S536) expression was normalized to total p65 protein levels. (**D**) The cultured medium from cells treated with 1 ng/mL TNF-α for 30 min, followed by ADM for 5.5 h, was used to measure soluble ICAM-1 expression levels by ELISA. Statistical analysis was performed using one-way ANOVA. The graphs are presented as the means ± standard deviation (SD) from three independent experiments. * *p* < 0.05, ** *p* < 0.01, and *** *p* < 0.001.

To determine the impact of ADM on anti-inflammatory responses, HPAECs were pre-treated with TNF-α for 30 min prior to ADM treatment. We found that ADM significantly decreased the ICAM-1 expression and partially blocked the degradation of IκBα, an inhibitory enzyme that reduces the NF-κB activation, after TNF-α challenge (Figure 2A,B). Consistently, treatment with ADM reduced the phosphorylation of p65 at S536, a subunit of NF-κB (Figure 2C), indicating that ADM mitigates endothelial inflammation, at least in part, through inhibition of NF-κB signaling. Moreover, ADM treatment led to a significant reduction in the soluble ICAM-1, especially at 100 ng/mL (Figure 2D). Additionally, ADM treatment reduced phosphorylation of p65, and blocked degradation of IκBα after LPS challenge (Supplementary Figure S1), consistent with the finding with TNF-α. Together, these results demonstrate that ADM exerts potent anti-inflammatory effects in endothelial cells exposed to TNF-α and LPS, likely via suppression of NF-κB activation.

### 3.3. ADM Improves Endothelial Homeostatic Markers Under Shear Stress

**Figure 3:**
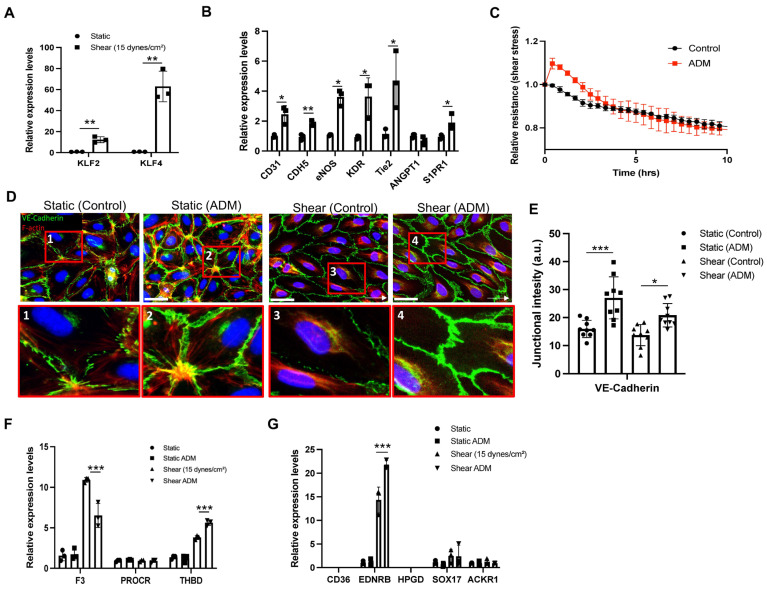
ADM and physiological shear stress synergistically improve endothelial characteristics. (**A**,**B**) HPAECs were incubated under unidirectional flow at 15 dynes/cm^2^ for 1 day. The expressions of mechanosensory genes (*KLF2* and *KLF4*) and pan-endothelial cell marker genes (*CD31*, *CDH5*, *NOS3*, *KDR*, *TEK*, *ANGPT1*, and *S1PR1*) were amplificated by real time PCR, normalized to *GAPDH*, and presented as graphs. Statistical analysis was performed using *t*-test. (**C**) The cell barrier function was measured by ECIS assay. The graph shows the results from the cells treated with 100 ng/mL ADM after 1 day of shear stress at 15 dyne/cm^2^. (**D**) Immunofluorescent images of F actin (red), and VE-Cadherin (green) were taken after the cells were treated with 100 ng/mL of ADM for 1 h under both static and shear stress conditions. For shear stress condition, the cells were incubated for 1 day at 15 dynes/cm^2^ before the ADM treatment. (**E**) The fluorescent intensity of junctional VE-Cadherin was measured, and statistical analysis was performed using one-way ANOVA. Each dot indicates one measurement. (**F**,**G**) The cells were incubated with either static or shear stress condition for 1 day, and then 100 ng/mL of ADM was added to the cells after medium change for 30 min. The indicated gene expressions were measured by real-time PCR and normalized to GAPDH. Statistical analysis was performed using two-way ANOVA. The graphs are presented as the means ± standard deviation (SD) from three independent experiments. White scale bars: 50 μm, * *p* < 0.05, ** *p* < 0.01, and *** *p* < 0.001.

Pulmonary endothelial cells are continuously exposed to hemodynamic forces that are essential for maintaining cellular phenotype and physiological functions. To examine the effects of shear stress on endothelial behavior, we applied unidirectional laminar flow using an Ibidi system. After 48 h of exposure to a shear stress of 15 dynes/cm^2^, HPAECs exhibited increased expression of the mechanotransduction transcription factors *KLF2* and *KLF4*, along with upregulation of canonical endothelial markers *CD31*, *CDH5*, *NOS3*, *KDR*, *TEK*, and *S1PR1* (Figure 3A,B), suggesting an improvement of endothelial characteristics and homeostasis.

To further assess the effect of ADM on endothelial barrier function under shear stress, ADM (100 ng/mL) was added on day 2 of flow culture, and transendothelial electrical resistance was monitored over 10 h. Consistent with the findings under static culture, ADM markedly enhanced endothelial cell barrier function under shear conditions (Figure 3C). The reorganization of actin cytoskeleton and adherens and tight junction proteins is closely linked to cell–cell junction regulation [15]. Immunofluorescence images showed that cells under static culture were randomly oriented, with F-actin filaments located at both the cell borders and the perinuclear region, whereas under shear conditions, the cell morphology was changed, and F-actin filaments were reorganized in the direction of flow and significantly diminished at the cell borders (Figure 3D), suggesting the significant impact of shear stress on actin cytoskeleton reorganization. ADM treatment further reorganized VE-cadherin along the cell borders, promoting a more linear and thicker arrangement that led to the structural reinforcement and stabilization of cell–cell junctions in both shear and static conditions (Figure 3D). Notably, although VE-cadherin staining at cell–cell borders was significantly increased following ADM treatment in both conditions (Figure 3E), total VE-cadherin expression levels remained unchanged (Supplementary Figure S2). These findings suggest that ADM reinforces endothelial barrier function under shear stress, primarily through cytoskeletal remodeling and stabilization of adherens junctions.

We further assessed the impact of ADM on the expression of endothelial homeostasis, inflammation, and coagulation-related gene expression under shear conditions. We found that ADM had modest or no impact on the improved level of endothelial markers such as *TEK*, *ANGPT1*, and *S1PR1* (Supplementary Figure S3A) under shear conditions. Shear stress at 15 dynes/cm^2^ alone modestly changed the level of pro-inflammatory markers including *IL1B*, *IL6*, and *ICAM1* (Supplementary Figure S3B), but strongly affected coagulation-related genes. Shear stress increased coagulation factor III (*F3*; a pro-coagulant factor) and thrombomodulin (*THBD*; an anti-coagulant enzyme) (Figure 3F). ADM treatment shifted this balance toward an anti-coagulative state by decreasing *F3* and increasing *THBD* (Figure 3F), suggesting that while shear stress regulates pro- and anti-coagulant balance, ADM promotes an anti-coagulation phenotype. To further determine whether ADM and shear stress had a synergistic effect on endothelial phenotypes, we performed a two-way ANOVA. This analysis revealed a significant interaction between shear stress and ADM treatment on *THBD* gene expression (*p* = 0.001), indicating a synergistic effect of ADM and shear stress on *THBD* regulation.

It is well known that endothelial cells (ECs) tend to lose their native, tissue-specific phenotypes and adopt more generic characteristics when cultured under static in vitro conditions using standard commercial media [8,16]. We therefore evaluated the impact of ADM and shear stress on endothelial phenotypic markers [8,17]. Specifically, we assessed the canonical markers for EC subpopulation in the lung vasculature, including *EDNRB* and *HPGD* for aCap, *CD36* for gCap, *SOX17* for arterial, and *ACKR1* for venous endothelial cells. Shear stress alone significantly increased the expression of *EDNRB*, while modestly increased on *SOX17* and had no impact on *ACKR1*. Notably, ADM treatment for 24 h under shear conditions synergistically enhanced *EDNRB* expression (Figure 3G), as indicated by a significant interaction between ADM and shear stress in a two-way ANOVA (*p* = 0.0027), with no impact on other genes. These results suggest that shear stress alone can partially restore endothelial subtype-specific gene expression, while ADM further amplifies this effect, particularly in aCap-associated markers. Collectively, these findings indicate that shear stress promotes endothelial homeostasis and specification, and that ADM further enhances this process by favoring an anticoagulant endothelial state and upregulating EDNRB.

### 3.4. ADM Regulates Endothelial Cell Functions Through an RAMP2-Dependent Mechanism

**Figure 4:**
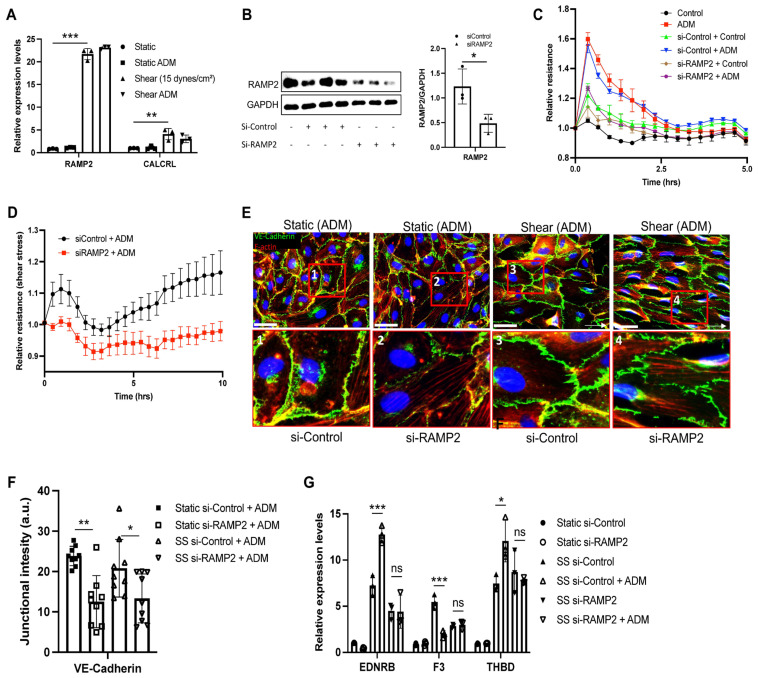
Knockdown of RAMP2 attenuates ADM-mediated enhancement of barrier function and normalization of pulmonary EC phenotype. (**A**) mRNA expression of ADM target receptors, including RAMP2 and CALCRL, was measured under static and shear stress conditions in the presence of ADM treatment. (**B**) To knock down of RAMP2 gene expression, control or RAMP2 siRNA (10 nM) were transfected into HPAECs. The ability of ADM (100 ng/mL) to enhance endothelial cell barrier function was measured under both (**C**) static and (**D**) shear stress (15 dyne/cm^2^) conditions. (**E**) Immunofluorescent images of F actin (red), and VE-Cadherin (green) were captured from the cells treated with ADM (100 ng/mL) for 1 h, under both static and shear stress (15 dyne/cm^2^) conditions after transfection of control or RAMP2 siRNA. (**F**) The fluorescent intensity of junctional VE-Cadherin was measured, and statistical analysis was performed using one-way ANOVA. Each dot indicates one measurement. (**G**) The genes *EDNRB*, *F3*, and *THBD*, which are regulated by ADM treatment under shear stress, were assessed in the RAMP2 knockdown condition. Statistical analysis was performed using one-way ANOVA or two-way ANOVA. The graphs are presented as the means ± standard deviation (SD) from three independent experiments. * *p* < 0.05, ** *p* < 0.01, and *** *p* < 0.001.

To further determine the signaling regulation of ADM on endothelial function, we first examined the expression levels of ADM receptor under both static and shear conditions. Interestingly, receptor activity-modifying protein (RAMP2) and calcitonin receptor-like receptor (CALCRL), co-receptors for ADM, were significantly increased under flow, with RAMP2 increasing the most (Figure 4A). ADM treatment did not alter the expression of either RAMP2 or CALCRL. To further determine the impact of RAMP2 on ADM-induced endothelial maturation, RAMP2 was knocked down with siRNA (Figure 4B), followed by ADM treatment. We found that ADM’s impact on endothelial cell barrier protection was almost completely abolished under both static and shear stress conditions (Figure 4C,D). Furthermore, the ADM-induced VE-cadherin reorganization along the cell borders was also eliminated after RAMP2 depletion (Figure 4E,F). The regulation of *EDNRB*, *F3*, and *THBD* gene expression by ADM under shear stress was reduced in RAMP2 knockdown cells (Figure 4G). Thus, these data demonstrate that ADM-induced effects are dependent on RAMP2, in which the expression level is enhanced by shear stress. These data further suggest that basal RAMP2 levels under static conditions are insufficient to support robust ADM signaling.

## 4. Discussion

Cellular components such as the endothelium in current in vitro vascular models, including organoids, organ-on-chip, organoid-on-chip systems, and human ex vivo tissue cultures, often exhibit an activated phenotype characterized by reduced barrier integrity and elevated inflammatory marker expression, limiting their ability to accurately recapitulate physiological vascular functions. One major contributing factor is that these models are typically maintained in media supplemented with a limited set of growth factors, such as VEGF-A and FGF, or with serum, which contains heterogeneous components derived from clotted blood [10,11,16]. These conditions fail to recapitulate the native cell–cell crosstalk and paracrine signaling required to maintain endothelial homeostasis.

In this study, we compared the effects of multiple soluble ligands previously identified within the human lung vascular niche on endothelial phenotype and function. We demonstrate that adrenomedullin (ADM) exerts a superior effect on enhancing endothelial barrier integrity compared with other ligands tested (Figure 1). Moreover, ADM attenuated inflammatory responses induced by TNF-α and LPS. Because shear stress is essential for maintaining vascular homeostasis and current in vitro vascular models commonly incorporate perfusion, we assessed the impact of ADM on endothelial phenotype and function under dynamic culture conditions. Under physiological shear stress (15 dynes/cm^2^), ADM preserved barrier enhancement, promoted anticoagulant properties, and reinforced native endothelial characteristics. Collectively, this work will provide instructive guidance for the development of optimized culture conditions by supplementing with native soluble factors, such as ADM, to promote vascular homeostasis/quiescence in vitro and improve the physiological fidelity of current bioengineered vascular model systems.

Leveraging scRNA-seq, we previously identified a group of ligand–receptor pairs that are enriched in the vascular niche in the healthy adult human lungs [8]. However, the impact of these factors on endothelial cell behaviors has not been studied. In the current study, we applied two different assays (ECIS and XperT) to compare the effects of different ligands on endothelial cell barrier function. Our data indicated that Ang-1 and ADM significantly decreased endothelial permeability in both assays, consistent with previous literature [18,19]. Interestingly, we observed that ADM had a significantly greater effect on improving the vascular barrier as compared to other molecules, which prompts us to focus on ADM in further studies. We further found that ADM reduced vascular inflammation following challenges with TNF-α and LPS. Although this anti-inflammatory effect was modest, it may reflect the ADM concentration used, which was optimized for barrier regulation and could be sub-optimal for detecting maximal anti-inflammatory responses. Previous studies have shown that higher concentrations of ADM can elicit stronger endothelial protective effects [20].

Physiological shear can stimulate endothelial homeostasis and reduce inflammatory and coagulation signals [21,22,23]. Under hemodynamic forces, especially laminar flow, quiescence and anti-inflammatory phenotypes are induced through multiple mechanotransduction pathways, including KLF2/KLF4, Nrf2, AMPK, and SIRT1 cascades [24,25,26,27,28]. After two days of dynamic flow culture using an ibidi microfluidic system, we observed significant upregulation of *KLF2* and *KLF4*. Pan-endothelial markers including *CD31*, *CDH5*, *NOS3*, *KDR*, *TEK*, and *S1PR1* were also markedly increased (Figure 3B), consistent with improvement of a quiescent endothelial phenotype [29,30,31]. Hemodynamic forces could also activate mechanosensitive channel PIEZO1, leading to ADM release from endothelial cells [32,33]. ADM subsequently activates CALCRL in complex with RAMP2, triggering Gs-cAMP-PKA signaling that both attenuates NF-κB-dependent mechanism [33] to reduce inflammation and phosphorylates eNOS at Ser633/635 and S1177 to resolve blood clot formation, thereby maintaining endothelial homeostasis [32]. Together, these findings suggest a potential interplay in which shear stress induces ADM signaling to reinforce endothelial quiescence and vascular homeostasis. Future studies will be required to directly dissect the molecular crosstalk between shear stress-mediated mechanotransduction and ADM signaling pathways.

Pulmonary vascular diseases, including sepsis, pneumonia, and pulmonary hypertension, are characterized by inflammation and activation of the coagulation cascade [34,35,36]. Low or absent shear promotes the formation of red blood cell (RBC)- and fibrin-rich (“red”) thrombi, whereas higher-shear arterial condition favor platelet-rich (“white”) thrombi [34,37,38]. Thrombomodulin is a transmembrane glycoprotein expressed on endothelial cells and serves as a cofactor for thrombin-mediated activation of protein C to APC, which has anticoagulant, anti-inflammatory, and anti-apoptotic effects [39]. Previous study demonstrates that laminar flow enhanced THBD expression in human brain microvascular endothelial cells [40]. Consistently, our results show that physiological shear stress increased *THBD* expression, and modulated the coagulation profile, with an increase in *F3* (tissue factor), the initiator of the extrinsic coagulation pathway, while endothelial protein C receptor (*PROCR*) remained unchanged, suggesting that shear stress at 15 dynes/cm^2^ may regulate a balance between pro- and anti-coagulant states. In the presence of shear stress, ADM shifted the endothelial phenotype toward an anticoagulant state as evidenced by increased THBD and decreased F3, whereas no such effect was observed under static conditions (Figure 3E and Figure 4E). Cyclic AMP signaling has been reported to regulate coagulation and inflammation by downregulating F3 and upregulating THBD across monocytes, cancer, vascular smooth muscles, and endothelial cells, and this signal has been shown to be regulated through ADM-RAMP2 signaling [41,42,43,44,45]. In line with this, RAMP2 knockdown abrogated ADM-induced modulation of F3 and THBD (Figure 4E), consistent with engagement of the ADM-CALCRL/RAMP2-cAMP pathway.

There are two major endothelial cell populations within lung microvascular bed, aCap, which are specialized for gas exchange and immune cell recruitment, and gCap, which regulate vasomotor tone and serve as progenitors in the lung microvasculature [17]. We found that shear stress alone (15 dynes/cm^2^) could markedly increase *EDNRB* expression, a critical marker for aCap cells, and this effect was further enhanced by ADM treatment. Both hemodynamic forces and ADM have been reported as critical cues contributing to vasodilation in the lung vasculature. EDNRB is known to mediate vasodilation upon binding with endothelins (ET-1, ET-2 and ET-3) [46]. Previous studies have reported that ADM enhances ET-1 release and promotes ET-1-EDNRB interactions in rat lung endothelial cells [47]. Together, these results suggest that ADM might synergistically enhance vasodilatory responses through an ET-1/EDNRB-dependent mechanism under shear stress.

There are several limitations associated with this project: (1) We primarily compared the effects of different ligands on endothelial barrier function and subsequently focused on ADM for detailed analysis. This prioritization was based on ADM’s strong and reproducible effects on barrier integrity, which suggested a robust biological role. As a result, other ligands were not comprehensively evaluated for their anti-inflammatory, anticoagulant, or native endothelial phenotype-related effects. Future studies will systematically assess these additional functional dimensions. (2) Another limitation is that our shear stress experiments were conducted at a single physiological condition (15 dynes/cm^2^), chosen based on prior literature describing typical physiological shear levels. While this approach allowed us to directly compare results with published findings, it limited our ability to examine potential dose- or pattern-dependent effects of shear stress. Subsequent studies will explore a broader range of shear conditions to better capture the mechanobiological spectrum of endothelial responses. (3) Additionally, in this study, we used HPAECs as a representative endothelial cell population for lung vascular biology to investigate the biological impact of different ligands. HPAECs are better characterized than other lung-derived endothelial cells; they are phenotypically stable, highly proliferative, and widely used in pulmonary vascular research. In contrast, commercially available human pulmonary microvascular endothelial cells and pulmonary venous endothelial cells are often contaminated with lymphatic endothelial cells [8], which may significantly affect ligand-induced biological responses. In future studies, purified populations of pulmonary microvascular and venous endothelial cells will be obtained by removing Prox1^+^ lymphatic endothelial contamination to further characterize the biological functions of soluble ligands in additional lung endothelial cell populations.

## 5. Conclusions

Together, our data support that ADM responsiveness is shear-dependent, in which shear enables robust effects, whereas static condition does not, underscoring shear’s role as an integrator of mechanotransduction and paracrine inputs. Whether ADM’s shear-dependent effects persist across different shear stress magnitudes and endothelial subtypes remains to be determined, a question with direct implications for how we model endothelial homeostasis and test soluble cues in vitro.

## Figures and Tables

**Table 1:** Primer list.

Gene	Sequence (5-3)		Size	NCBI Reference
KLF2	Forward	ACTTTCGCCAGCCCGTGC	103	NM_016270.4
	Reverse	AGTCCAGCACGCTGTTGAG		
KLF4	Forward	CTGCGGCAAAACCTACACAA	182	NM_001314052.2
	Reverse	GGTCGCATTTTTGGCACTG		
CD31	Forward	CCAGTGTCCCCAGAAGCAAA	81	NM_000442.5
	Reverse	TGATAACCACTGCAATAAGTCCTTTC		
CDH5	Forward	TTGGAACCAGATGCACATTGAT	86	NM_001795.5
	Reverse	TCTTGCGACTCACGCTTGAC		
NOS3	Forward	GCCGGAACAGCACAAGAGTTAT	150	NM_000603.5
	Reverse	AGCCCGAACACACAGAACC		
KDR	Forward	CGGTCAACAAAGTCGGGAGA	123	NM_002253.4
	Reverse	CAGTGCACCACAAAGACACG		
TEK	Forward	GATTTTGGATTGTCCCGAGGTCAAG	327	NM_000459.5
	Reverse	CACCAATATCTGGGCAAATGATGG		
ANGPT1	Forward	TGGCTGCAAAAACTTGAGAATTAC	145	NM_001146.5
	Reverse	TTCTGGTCTGCTCTGCAGTCTG		
S1PR1	Forward	TGCGGGAAGGGAGTATGTTT	198	NM_001400.5
	Reverse	TGCAGTTCCAGCCCATGATA		
F3	Forward	CAGACAGCCCGGTAGAGTGT	75	NM_001993.5
	Reverse	CCACAGCTCCAATGATGTAGAA		
PROCR	Forward	ACTTCTCTTTTCCCTAGACTGC	169	NM_006404.5
	Reverse	TGAAGTCTTTGGAGGCCATCT		
CD36	Forward	ACTGAGGACTGCAGTGTAGG	223	NM_001001548.3
	Reverse	GGTTTCTACAAGCTCTGGTTCTTA		
EDNRB	Forward	CTGGCCATTTGGAGCTGAGA	200	NM_000115.5
	Reverse	CCAGAACCACAGAGACCACC		
HPGD	Forward	CATGCACGTGAACGGCAAAG	155	NM_000860.6
	Reverse	GCTCATCCAGGGCAGCTTTA		
SOX17	Forward	AAGGGCGAGTCCCGTATC	221	NM_022454.4
	Reverse	TTGTAGTTGGGGTGGTCCTG		
ACKR1	Forward	GATGGCCTCCTCTGGGTATG	198	NM_001122951.3
	Reverse	AAGGGCAGTGCAGAGTCATC		
RAMP2	Forward	CTGTCCTGAATCCCCACGAG	191	NM_005854.3
	Reverse	CAGGGTGCTATAAGGCCTGC		
CALCRL	Forward	AAGAGCTGGACTGGGTCTTGA	212	NM_005795.6
	Reverse	GAAGTTTGCAGCAGTCTTGTCA		
THBD	Forward	CCCAACACCCAGGCTAGCT	76	NM_000361.3
	Reverse	CGTCGATGTCCGTGCAGAT		
GAPDH	Forward	ATGGGGAAGGTGAAGGTC	108	NM_002046.7
	Reverse	GGGGTCATTGATGGCAACAATA		
IL1B	Forward	CAGGCTGCTCTGGGATTCTC	172	NM_000576.3
	Reverse	GTCCTGGAAGGAGCACTTCAT		
IL6	Forward	ACTCACCTCTTCAGAACGAATTG	149	NM_000600.5
	Reverse	CCATCTTTGGAAGGTTCAGGTTG		
ICAM1	Forward	ACCATCTACAGCTTTCCGGC	293	NM_000201.3
	Reverse	CAATCCCTCTCGTCCAGTCG		

## Data Availability

The original contributions presented in the study are included in the article, further inquiries can be directed to the corresponding author.

## Supplementary Materials

The following supporting information can be downloaded at https://www.mdpi.com/article/10.3390/cells15020152/s1, Figure S1: ADM attenuates pro-inflammatory effect of LPS. HPAECs were treated with ADM at 100 ng/mL for 30 min, followed by LPS (50 ng/mL) for 6 h. The samples were subjected to WB analysis to detect IκBα, phosphorylated-p65 (S536), total p65, and GAPDH. A) IκBα proteins were normalized to GAPDH, and the values were presented as a bar graph. (B) Phosphorylated-p65 (S536) expression was normalized to total p65 protein levels. The graphs are presented as the means ± standard deviation (SD) from three independent experiments. ***** *p* < 0.05; Figure S2: ADM and *CDH5* expression under shear stress. The cells were incubated under either static or shear stress condition for 1 day, followed by the treatment of ADM. The expression level of *CDH5* was evaluated. The graphs are presented as the means ± standard deviation (SD) from three independent experiments. ***** *p* < 0.05.; Figure S3: ADM and endothelial characteristics under shear stress. (A and B) The cells were incubated under either static or shear stress condition for 1 day, followed by the treatment of ADM. The samples were collected after 24 h of treatment. The expression level of *ANGPT1*, *S1PR1*, *TEK*, *IL1B*, *IL6*, and *ICAM1* were evaluated. The graphs are presented as the means ± standard deviation (SD) from 3–5 independent experiments. ns indicates non-significant.
