# Supplementary material for: Adrenomedullin-RAMP2 Enhances Lung Endothelial Cell Homeostasis Under Shear Stress
Source: Cells. 2026 Jan 14;15(2):152. doi: 10.3390/cells15020152 (PMC12839701; doi:10.3390/cells15020152)
Supplement: Supplementary file 1 [file cells-15-00152-s001.zip › cells-4026591-supplementary.pdf]

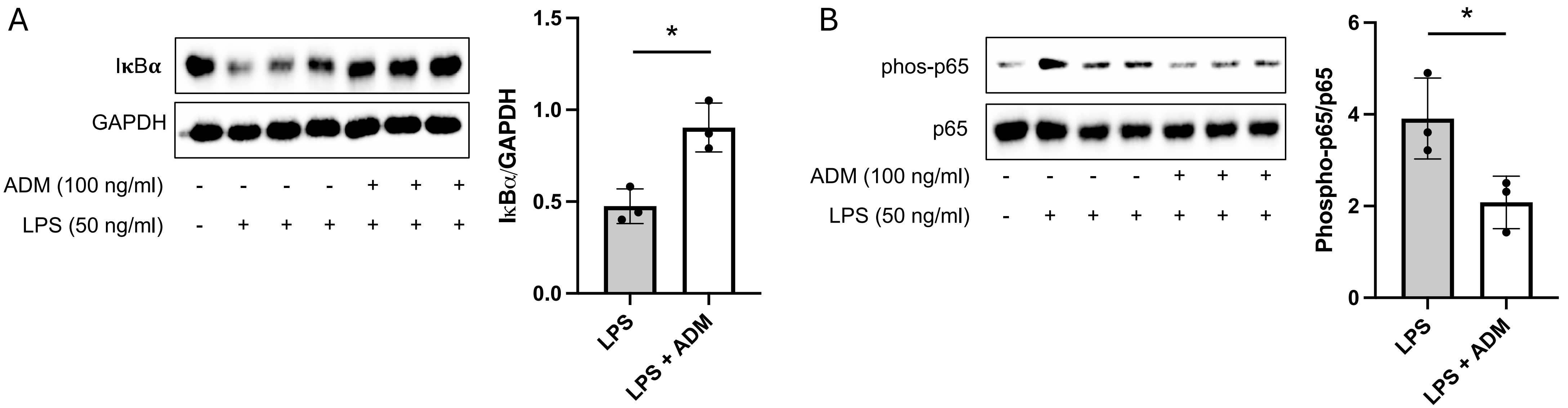


**Supplemental Figure S1. ADM attenuates pro-inflammatory effect of LPS.** HPAECs were treated with ADM at 100 ng/mL for 30 min, followed by LPS (50 ng/ml) for 6 hours. The samples were subjected to WB analysis to detect IκBα, phosphorylated-p65 (S536), total p65, and GAPDH. A) IκBα proteins were normalized to GAPDH, and the values were presented as a bar graph. (B) Phosphorylated-p65 (S536) expression was normalized to total p65 protein levels. The graphs are presented as the means ± standard deviation (SD) from three independent experiments. *$p<0.05$.

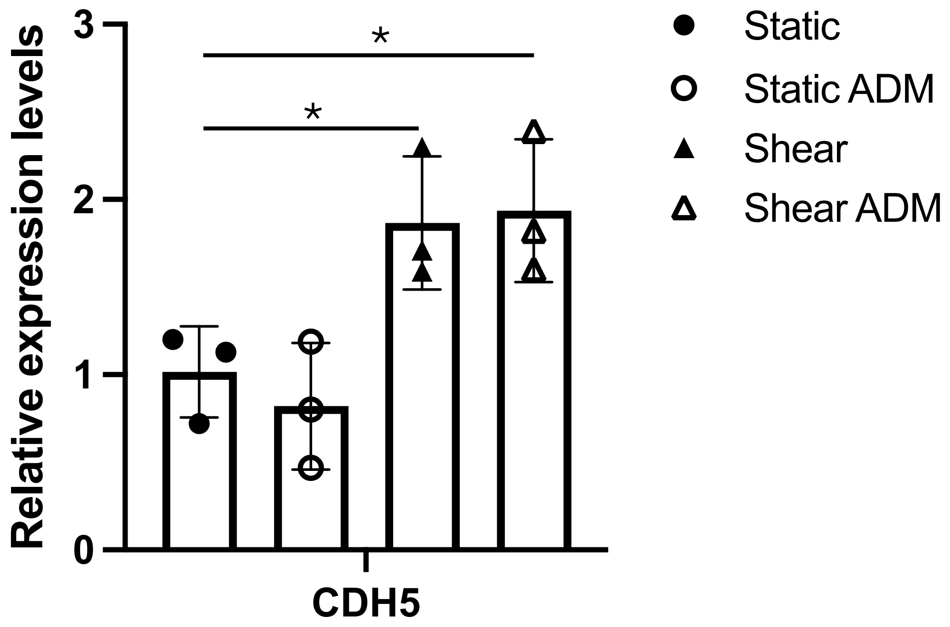


**Supplemental Figure S2. ADM and *CDH5* expression under shear stress.** The cells were incubated under either static or shear stress condition for 1 day, followed by the treatment of ADM. The expression level of *CDH5* was evaluated. The graphs are presented as the means ± standard deviation (SD) from three independent experiments. *****$p<0.05$.

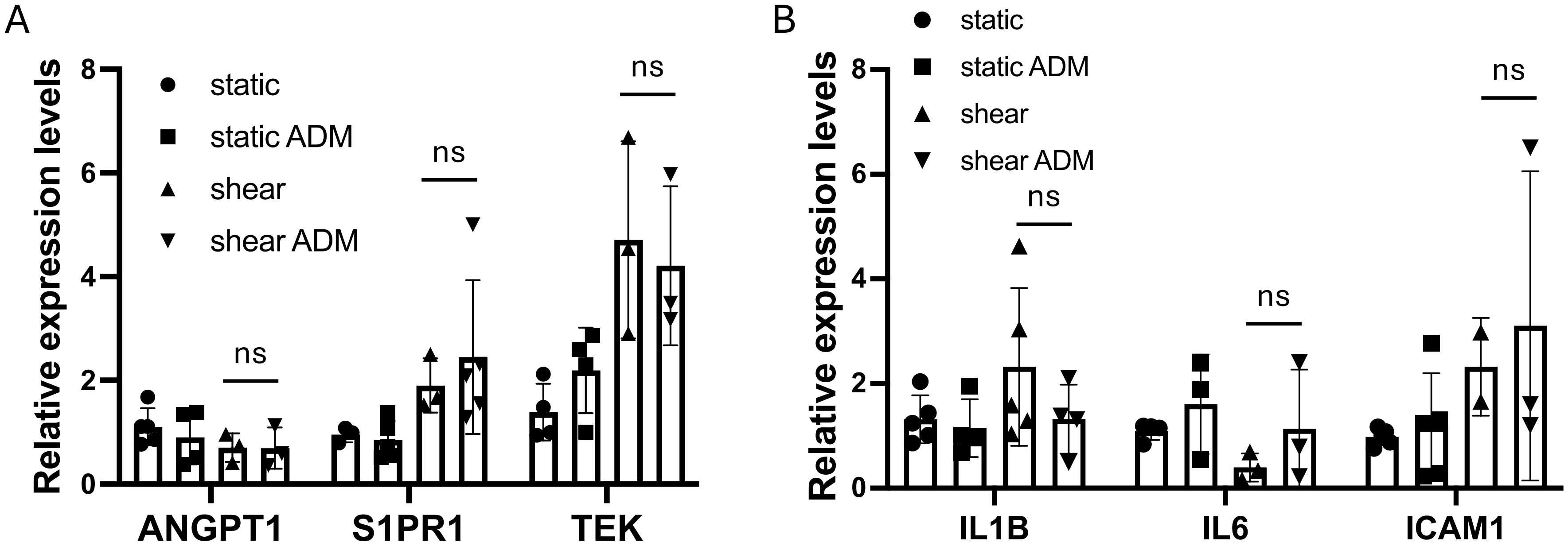


**Supplemental Figure S3. ADM and endothelial characteristics under shear stress.** (A and B) The cells were incubated under either static or shear stress condition for 1 day, followed by the treatment of ADM. The samples were collected after 24 hours of treatment. The expression level of *ANGPT1*, *S1PR1*, *TEK*, *IL1B*, *IL6*, and *ICAM1* were evaluated. The graphs are presented as the means ± standard deviation (SD) from 3 - 5 independent experiments. ns indicates non-significant.
